# Diabetes Mellitus Is Associated with Hepatocellular Carcinoma: A Retrospective Case-Control Study in Hepatitis Endemic Area

**DOI:** 10.1371/journal.pone.0084776

**Published:** 2013-12-26

**Authors:** Ze Zheng, Chao Zhang, Jianhua Yan, Yanping Ruan, Xiaoyi Zhao, Xingting San, Yilei Mao, Qinghua Sun, Kezhong Zhang, Zhongjie Fan

**Affiliations:** 1 Department of Cardiology, Peking Union Medical College Hospital, Chinese Academy of Medical Sciences & Peking Union Medical College, Beijing, China; 2 Division of Cardiovascular Medicine, Davis Heart & Lung Research Institute, College of Medicine, Ohio State University, Columbus, Ohio, United States of America; 3 Division of Environmental Health Sciences, College of Public Health, Ohio State University, Columbus, Ohio, United States of America; 4 Center for Molecular Medicine & Genetics, Wayne State University School of Medicine, Detroit, Michigan, United States of America; 5 Department of Hepatology, Peking Union Medical College Hospital, Chinese Academy of Medical Sciences & Peking Union Medical College, Beijing, China; 6 Department of Immunology and Microbiology, Wayne State University School of Medicine, Detroit, Michigan, United States of America; University of the Witwatersrand, South Africa

## Abstract

**Background:**

A number of case-control patient studies have been conducted to investigate the association between diabetes mellitus (DM) and hepatocellular carcinoma (HCC). Despite some controversial reports, it has been suggested that DM is associated with HCC. The previous studies on this subject vary in the selection of populations, sample sizes, methodology, and analysis results. Therefore, it is necessary to further delineate the involvement of DM, together with other related risk factors, in HCC with large sample size and strict analysis methodology.

**Methods:**

We conducted a hospital-based retrospective case-control study at Perking Union Medical College Hospital, China. A total of 1,568 patients with liver diseases were enrolled in the statistical study to evaluate the association of DM and other risk factors with HCC. Among these patients, 716 of them were diagnosed with benign liver diseases, and 852 patients were diagnosed as HCC. We utilized binary logistic regression and stepwise logistic regression to investigate the associations among DM, hypertension, fatty liver, cirrhosis, gallstone, HBV infection, HCV infection, and HCC.

**Results:**

Statistical analysis through the stepwise regression model indicated that the prevalence of DM, male gender, cirrhosis, HCV infection, or HBV infection is higher in the HCC patient group compared to the control group. However, the prevalence of gallstone is negatively associated with HCC cases. DM co-exists with HBV infection, male gender, and age in the HCC cases. Binary logistic regression analysis suggested that DM may synergize with HBV infection in HCC development.

**Conclusion:**

DM is strongly associated with the increased risk of HCC regardless of the prevalence of HBV infection, HCV infection, cirrhosis, male gender, and age. However, the synergistic interaction between DM and HBV in HCC occurrence is significant. Therefore, DM patients with HBV infection represent a very high HCC risk population and should be considered for HCC close surveillance program.

## Introduction

Hepatocellular carcinoma (HCC) is one of the most common cancers worldwide [1]. Infection with hepatitis B (HBV) and/or hepatitis C (HCV) viruses and elevated alcohol consumption are the leading risk factors for HCC [2,3]. Increasing evidences suggested metabolic syndromes, such as diabetes mellitus (DM) and non-alcoholic fatty liver diseases (NAFLD) have strong association with the incidence of HCC [2,4]. China is a hepatitis and HCC endemic area, accounting for over 50% of HCC cases worldwide. In China, over 70% of HCC cases have been linked to hepatitis B virus (HBV) infection. 

A number of cohort and case-control studies have investigated the relationship between diabetes mellitus (DM) and HCC risk [4,5]. Although there were some controversial reports [6], DM has been suggested as a risk factor for the development of HCC. However, the involvement of DM in the development of HCC in the presence of other risk factors, such as HBV, hypertension, fatty liver, and cirrhosis, has not been well documented. Only a few cohort studies have followed populations with both chronic HBV infection and metabolic disorders. Most of the reported case-control studies used a normal population or cancers as controls, and liver pathology and metabolic disorders were not well matched between cases and controls. Additionally, it has been speculated that DM may synergize with other etiological risk factors, such as age, male gender, and metabolic dysregulation, in driving HCC.

In this study, we conducted a hospital-based case-control study to more precisely investigate the association between DM and HCC in the presence of other risk factors with large size of samples and strict multiple analysis methodology. We concluded that DM represents an independent or synergizing risk factor with HBV, age, or male gender for HCC development in Chinese population.

## Patients and Methods

### Ethics Statement

We conducted a retrospective case-control study on liver disease based on the liver disease patients in the Peking Union Medical College Hospital, Beijing, China, from May, 2008 to September, 2012. This study was approved by the Institutional Review Board (IRB) committee of Peking Union Medical College Hospital and Peking Union Medical College (approval documents attached). Written consent given by the patients was waived by the approving IRB.

### Study Subjects

Cases were patients <70 years of age with benign liver diseases or HCC that went through surgeries. The patients were from 29 out of 34 provinces in China. The case selection criteria include: the patients have full blood testing information; the first time patients (the returning patient cases were removed from the study); and the HCC patients had yet received any cancer treatment at study entry. In total, 1568 participants had complete diagnostic records. Among all these patients, 716 patients were diagnosed with benign liver diseases, and 852 patients were diagnosed with HCC. Among these HCC patients, there were 603 men (70.8%) with a mean age of 57.49 ± 11.86 years; 362 patients (42.5%) were positive for HBsAg; and 37 patients (4.3 %) were positive for anti-HCV (Table 1).

**Table 1:** Comparison of Risk Factors and Demographics of the HCC and Controls Groups.

Variable	Controls (n=716)		HCC (n=852)		χ^2^	p
	Frequency	Percent	Frequency	Percent		
Male	221	30.9	603	70.8	248.5	<0.001
Cirrhosis	15	2.1	271	31.8	230.3	<0.001
HBsAg**^*+*^**	34	4.7	362	42.5	293.5	<0.001
Anti-HCV**^*+*^**	5	0.7	37	4.3	19.8	<0.001
Portal hypertension	8	1.1	39	4.6	16.0	<0.001
Diabetes	30	4.2	90	10.6	22.4	<0.001
Gallstone	118	16.5	93	10.9	10.4	0.001
Fatty liver	29	4.1	17	2.0	5.8	0.016
Hypertension	118	16.5	167	19.6	2.6	0.110

HCC, hepatocellular carcinoma; HBsAg, hepatitis B surface antigen; HCV, hepatitis C virus.

### Controls

Among the liver disease patients, 716 patients diagnosed with benign liver diseases were used as the control. Of these, 34 (4.7%) were positive for HBsAg, and 5 (0.7 %) were positive for anti-HCV (Table 1).

### Study variables

The variables analyzed in this study included age, sex, HBsAg, anti-HCV, alanine transaminase (ALT), aspartate aminotransferase (AST), glucose, plasma lipids, inflammatory markers, and DM (Table 2). DM was defined based on 1) fasting plasma glucose 126 mg/dl or above, 2) oral glucose tolerance test 200 mg/dl or above, or 3) A1C abnormality (http://diabetes.niddk.nih.gov/dm/pubs/diagnosis/) [7].

**Table 2:** Comparison of Blood Risk Factors between the HCC and Controls Groups.

Variable	Controls (Mean ±SD/ Median Q1-Q3)	HCC (Mean ±SD/ Median Q1-Q3)	t	p
Age (year)	48.9 ± 13.1	57.5 ± 11.9	-13.45*	<0.001
ALT (IU/L)	17.0 (12.0, 25.0)**^*#*^**	29.0 (18.0, 52.0)**^*#*^**	-7.76*	<0.001
AST (IU/L)	20.0 (17.0, 24.0)**^*#*^**	32.0 (22.0, 48.5)**^*#*^**	-11.01*	<0.001
GGT (IU/L)	23.0 (15.0, 41.0)**^*#*^**	51.0 (26.0, 98.0)**^*#*^**	-6.95*	<0.001
ALP (IU/L)	61.0 (49.0, 81.0)**^*#*^**	82.0 (63.0, 108)**^*#*^**	-5.85	<0.001
LDH (IU/L)	162.1 ± 68.1	194.0 ± 93.7	-7.68*	<0.001
ChE (U/mL)	7.2 ± 1.6	6.0 ± 1.9	13.1*	<0.001
Glucose (mM)	4.9 ± 0.8	5.4 ± 1.6	-8.22*	<0.001
TBA (µmol/L)	3.8 (2.3, 6.3)**^*#*^**	6.5 (3.9, 12.8)**^*#*^**	-7.29*	<0.001
BU (µmol/L)	12.7 (10.0,16.5)**^*#*^**	14.0 (10.4, 19.1)**^*#*^**	-4.34*	<0.001
BC (µmol/L)	3.8 (3.0, 5.2)**^*#*^**	4.7 (3.5, 6.8)**^*#*^**	-4.13*	<0.001
TG (mmol/L)	1.3 ± 0.9	1.2 ± 0.8	1.56*	0.120

* Unequal variances; # Median and Quartile; Q1: the first quartile; Q3: the third quartile;

SD, standard deviation of the mean.HCC, Hepatocellular Carcinoma; ALT, alanine aminotransferase; AST, aspartate aminotransferase; GGT, glutamyltransferase; BU, total bilirubin; BC, direct bilirubin; ALP, alkaline phosphatase; TBA, total bile acid; LDH, lactate dehydrogenase;ChE, cholinesterase; TG, triglyceride;

### Statistical Analysis

The distribution of the study variables was calculated using means with standard deviations for normal continuous variables or using median with quartile range for skewness variables, and frequencies and percent for categorical variables. Univariate comparisons were performed using the chi-square test for categorical variables; for continuous variable comparisons, Student’s t tests were used when equality of variances was satisfied, otherwise Satterthwaite-tests were conducted. We assessed effect of each “risk” factor for HCC using binary logistic regression, i.e., unadjusted model. Stepwise logistic regression was performed in this study to find the most parsimonious set of predictors that are most effective in predicting HCC. The main ideal of stepwise logistic regression is that predictors are entered to the logistic regression model one at a time by reducing the -2Log Likelihood error for the included predictors. Each included predictors detected to see whether the model would be better than the predictors were excluded after each predictor is entered. The process of adding more variables stops when it is impossible to make a statistically significant reduction in -2Log Likelihood using any of the predictors not yet included. Since the exclude variables do not contribute to interpreting variance in the dependent variable, the predictive model is parsimonious and accurate. In doing stepwise logistic regression for this study, we choose entry value equals 0.25, and stay value is 0.10 respectively. It means that a significance level of 0.25 is required to allow a variable into the model, and a significance level of 0.10 is required for a variable to stay in the model. Leave-one-out cross-validation analysis was performed to estimate its effectiveness and performed with several statistical indexes, i.e., Overall Correct Classification Rates, Sensitivity, Specificity, and the Area under the Receiver Operating Characteristic (ROC) Curve.

Multi-collinearity frequently occurs when much more independent variables in a logistic regression mode is conducted. This problem can lead to unstable estimates and inaccurate variances that affect hypothesis test and confidence intervals. We used Spearman correlation coefficients to measure the degree of correlation between two quantitative variables, and used Contingency Coefficients to measure the degree of association between the two category variables. For dealing with this problem, our strategy was that the variables causing multi-collinearity were dropped from the model based on biomedicine and statistical background. It is high correlated between two independent variables if a coefficient of correlation is greater or equal 0.45 for this study. Statistical software SAS 9.3 was used for all model-fitting.

## Results

### Comparison of risk factors between HCC cases and their controls

The baseline demographic characteristics of patients and controls are summarized in Table 1. Most study subjects were Chinese; the men-to-women ratio was 1.11 to 1 for HCC patients. Case patients were slightly older than control subjects, with a mean difference of 8.5 years (Table 2). Comparison of blood risk factors between the HCC and controls groups indicates significant differences between these two groups on age, ALT, AST, GGT, ALP, LDH, ChE, glucose, TBA, BU, and BC (Table 2). However, there was no significant difference in TG levels between HCC and control groups. 

Table 3 shows odds ratios by binary logistic regression model. The risk factors that are strongly associated with HCC include: male gender (OR=5.42, p<0.0001), HBV infection (OR=14.8, p<0.0001), HCV infection (OR=6.46, p<0.0001), cirrhosis (OR=21.8, p<0.0001), DM (OR=2.7, p<0.0001), and portal hypertension (OR=4.25, p=0.0002). There is no significant correlation between Hypertension and HCC (OR=1.24, p=0.11). We found that risk factors that are negatively correlated with HCC include: ChE (OR=0.686, p<0.0001), Tch (OR=0.79, p=0.0002), HDL (OR=0.34, p<0.0001), Apoa1 (OR=0.29, p<0.0001), and gall stone (OR=0.62, p=0.0014). 

**Table 3:** Odds Ratios for Individual Effects of Predictors (Unadjusted Model) on HCC.

Variables	OR (95% CI)	p
Male	5.42 (4.37-6.74)	<0.001
Age	1.06 (1.05-1.07)	<0.001
HBsAg**^*+*^**	14.80 (10.20-21.50)	<0.001
Anti-HCV**^*+*^**	6.46 (2.52-16.51)	<0.001
ALT	1.02 (1.01-1.02)	<0.001
AST	1.05 (1.04-1.06)	<0.001
GGT	1.00 (1.00-1.01)	<0.001
ALP	1.00 (1.00-1.01)	<0.001
LDH	1.01 (1.01-1.01)	<0.001
ChE	0.69 (0.65-0.73)	<0.001
Glucose	1.51 (1.34-1.68)	<0.001
TBA	1.06 (1.04-1.08)	<0.001
BU	1.01 (1.01-1.02)	<0.001
BC	1.02 (1.01-1.03)	0.003
TG	0.88 (0.75-1.03)	0.110
Tch	0.79 (0.69-0.89)	<0.001
HDL	0.34 (0.23-0.52)	<0.001
LDL	0.81 (0.70-0.95)	0.008
APOA1	0.29 (0.17-0.48)	<0.001
APOB	0.59 (0.33-1.06)	0.076
LP(a)	0.99 (0.98-0.99)	0.011
hs-CRP	1.02 (1.00-1.03)	0.015
Cirrhosis	21.8 (12.8-37.1)	<0.001
DM	2.70 (1.76-4.14)	<0.001
Portal hypertension	4.25 (1.97-9.14)	<0.001
Gallstone	0.62 (0.46-0.83)	0.001
Fatty liver	0.48 (0.26-0.89)	0.019
Hypertension	1.24 (0.95-1.60)	0.110

We identified the effects of the independent variables associated with HCC by calculating the odds ratios through stepwise logistic regression model (Table 4). This model allowed us to identify the association among the variables based on the information of all patient cases (both control and HCC groups) enrolled. Note that it is very common that many variables exist in a high throughput data set, so multi-collinearity frequently occurs when statistical models are conducted. However, multi-collinearity can lead to unstable estimates and inaccurate variances that affect hypothesis test and confidence intervals [8]. For this study, we conducted Spearman correlation analysis to detect the degree of correction between two quantitative variables, and used Contingency Coefficients to measure the degree of correlation between two category variables. The correlation coefficients between ALT and AST, between ALT and GGT, between ALT and ALP were 0.835 (p<0.001), 0.639 (p<0.001), and 0.432 (p<0.001), respectively. The correlation coefficients between AST and ALP, LDH, and TBA were 0.481 (p<0.001), 0.462 (p<0.001), and 0.449 (p<0.001), respectively. It was 0.907 (p<0.001) between BU and BC. The Contingency Coefficients between HBV and Cirrhosis, between Portal hypertension and Cirrhosis, DM and Hypertension are 0.531 (p<0.001), 0.308 (p<0.001), and 0.214 (p<0.001), respectively. Our strategy for solving the problem of multi-collinearity in stepwise logistic regression model was that the variables causing multi-collinearity were dropped from the model so that the model was robust and interpretable. AST, GGT, ALP, BC, LDH, Cirrhosis, and Hypertension were dropped from independent variables before running stepwise logistic regression. The final model is the last step model, where adding another variable would not improve the model significantly. Table 4 shows the higher loading risk factors associated HCC to be included in the last step of the model. It may be seen that 8 out of 13 risk factors were found to be significant, based on the usual 0.05 significance level. There is no evidence of a lack of fit in the selected model according to the results of the Hosmer and Lemeshow test (χ^2^=10.27, p=0.24). Some form of validation analysis is a necessity when stepwise logistic regression is used. We used Leave-one-out cross-validation analysis to estimate its effectiveness and performance. Overall Correct Classification Rates, sensitivity, specificity, and the area under the ROC Curve were 80.1%, 78.9%, 81.4%, and 0.87, respectively. So the model explains the data well based on the statistical indexes above. 

**Table 4:** Odds Ratio from Stepwise Logistic Regression Model for HCC.

Variable	OR (95% CI)	P
Male	3.08 (2.36-4.02)	<0.001
Age	1.06 (1.05-1.07)	<0.001
HBsAg+	14.05(9.29-21.26)	<0.001
Anti-HCV**^*+*^**	4.61(1.60-13.19)	0.004
Diabetes	2.35 (1.36-4.05)	0.002
Gallstone	0.38 (0.25-0.56)	<0.001
ChE	0.78 (0.72-0.84)	<0.001
ALP	1.003 (1.001-1.005)	0.001

Models with covariates can detect the effects of each covariate while the effects of other covariates have been partialed out. Table 4 shows the results of the model predicting HCC by controlling for other covariates. P values are reported for the overall effect of each covariate controlling for the other covariates. In this model, HBV, HCV, gender, and DM were still strongly associated with HCC in the Chinese population. We confirmed that gallstone is negatively associated with HCC (OR=0.38, p<0.0001). Based on the comparison between DM positive and DM negative cases, there was an increase of 135% in odds of HCC. The values of odds ratio were trivially smaller (OR=14.05, OR=4.61, OR=3.08, and OR=2.35, respectively) than those in unadjusted model. The strong association between DM and HCC is consistent with cohort and case-control studies with the populations in US, Europe, and other Asian populations [9] [10-12] [13-16]. 

### Age is a confounding factor

The age difference between the disease (HCC) and control groups is statistical significant, as shown in Table 2. The years of age in the control and disease groups are 57.9 ± 11.9, and 48.9 ± 13.1, respectively. However, age acts as a confounding factor in this study. Age should meet three conditions as a confounder [17,18]: 1) it is a risk factor for HCC; 2) it is associated with DM; and 3) it is not in the causal pathway between DM and HCC. Our analysis indicated that age meets the first two conditions as shown in Tables 2-5. To detect the third condition, we used the Mantel-Haenszel method to takes into account the effect of the strata, “Younger” or “Older” for age. We changed age from continuous variable to binary variable to easily explain that age is a confounder, and set up the age value of 49 as cutoff based on its average of control group. It means that those who belong to “Younger” group are less than 49. Otherwise, the participants should be in “Older” group. As shown in Tables A-C (File S1), in the “Younger” group, ages in the control and HCC are 38.5 ± 8.6 and 41.7 ± 6.7, respectively; in the “Older” group, ages in control and HCC are 59.4 ± 7.2 and 62.5 ± 8.2, respectively. The odds ratio of entire samples, “Younger” group, and “Older” group are 2.7, 1.8, and 2.0, respectively. Both estimates (“Younger” and “Older”) of the odds ratios are lower than the odds ratio based on the entire samples. Additionally, the results of Breslow-Day Test for Homogeneity of the Odds Ratios (chi-square=0.038, p-value=0.845) indicate that the odds ratios on DM and HCC between the “Young” and “Older” groups are not significantly different in predicting the DM-HCC correlation between the “Young” and “Older” groups. 

**Table 5:** Odds Ratio from Adjusted Model by Controlling Age and Gender on HCC for Diabetes Cases (n=120).

Variable	Controls (n=30)		HCC (n=90)		OR (95% CI)	p
	Frequency or Mean	Percentor SD	Frequency or Mean	Percent or SD		
Age	60.2	10.5	63.7	9.1	1.05 (1.00-1.11)	0.053
Male	13	43.3	65	72.2	2.37 (0.95-5.94)	0.066
HBsAg**^*+*^**	1	3.3	26	28.9	12.77 (1.57-104.13)	0.017

### Potential synergizing risk factors with DM in HCC cases

Having established the positive correlation between DM and HCC by stepwise logistic regression analysis, we next defined the synergizing factors that may interact with DM in HCC development. For this purpose, we utilized binary logistic regression model to identify the risk factors that are positively correlated with HCC in DM cases (Table 5). Among these who were diagnosed with DM, the following risk factors are positively correlated with HCC (by comparing to the control): male gender (72.2% *vs* 43.3%), age, and HBV infection (28.9% *vs* 3.3%). Note that the p-values for male gender and age are slightly greater than 0.05. This is understandable, since DM doesn't have a strong gender and age prevalence like HCC. Interestingly, HCV infection, a leading HCC risk factor, did not interact with DM in HCC development in the patient cases we reviewed. This may be due to a potential limitation of this study, which includes a relatively small number of HCV patients. Taken together, our results implicate the importance of frequent screening HCC in DM patients in the HBV prevalent area, especially for those who had HBV infection. 

## Discussion

Increasing evidence has suggested the association between DM and HCC in different populations [9,10,13]. In our case-control study, both unadjusted logistic regression and stepwise logistic regression analysis indicated a strong and statistically significant association between DM and HCC in Chinese population where hepatitis is endemic. We showed that DM is strongly correlated with HCC development. DM co-coexists with HBV infection, male gender, and age in HCC development, suggesting the potential synergism among these risk factors in driving HCC. The evidence that DM is a risk factor of HCC that may work independently or synergistically with other risk factors, suggested by our study, is consistent with the previous studies with the populations in US [9,19-21], Europe [10-12], and Asian [13-16][22]. 

Our finding of a highly significant association between DM and HCC among the cases with or without hepatitis infection strengthens the notion that the DM-HCC association is direct in nature. The biological mechanism for the association between DM and HCC is not well understood. Elevation of serum insulin in DM patients represents a causative factor for the association between DM and HCC, although increased insulin level alone may not be sufficient in causing HCC. The development of HCC undergoes a long process in which hepatic pathogenesis leads to increased tissue turnover that eventually leads to the occurrence of HCC. In this perspective, non-alcoholic steatohepatitis (NASH) is an onset or manifestation of DM [23,24]. Cirrhosis, the established causative risk factor of HCC, is an advanced stage of NASH [23,25,26]. Therefore, NASH, the most common liver disease that can be caused by metabolic dysregulation, hepatic damage, or environmental stress [27-29], may account for another mechanism underlying the association between DM and HCC. Our study clearly showed the strong association of cirrhosis with HCC (Tables 3, 4). However, fatty liver, or hepatic steatosis, is inversely correlated with HCC in the patients we investigated (Table 3). The negative association of hepatic steatosis with HCC may be due to the nature of this retrospective case review study in which the patients with benign fatty liver diseases had been excluded from the HCC cases. 

The strength of our study is the large sample size and strict biostatical analysis methods. We assessed the effect of each “risk” factor for HCC using binary logistic regression, i.e., unadjusted model (Table 3). We used stepwise logistic regression to find the most parsimonious set of predictors that are most effective in predicting HCC among the independent variables (Tables 3-4). With this analysis model, we were able to identify independent correlations among the variables based on the information of all patients enrolled. In this study, the correlation between HCC and DM was given in the context of the associations among multiple risk factors, including DM, hypertension, fatty liver, cirrhosis, gallstone, HBV infection, HCV infection, and HCC. Logistic regression model was used to calculate the “adjusted” estimator, accounting for confounders. Models with covariates can detect the effects of each covariate while the effects of other covariates have been partialed out. Our study built adjusted stepwise logistic regression model to account for the effects of some of these variables. Table 4 shows the results of the model predicting HCC by controlling for other covariates. P values are reported for the overall effect of each covariate controlling for the other covariates. Our analysis results showed that the adjusted odds of HCC caused by DM are statistically significant. The adjusted odd is 2.35 by controlling for age, gender, HBsAg+, Gallstone, ChE, and ALP (Table 4). In another word, after the effects of age, gender, and HBsAg+ etc have been considered, the patients with diabetes more likely have HCC (with the odd value of 2.35). Some limitations are present in our study. It is a retrospective, hospital-based study drawn from clinical practice but not from the community. However, in this case-control study, we selected a control group that, for the known risk factors for HCC, could be considered representative population of liver disease in China. A potential bias in case-control studies is discerning the temporal relationship between risk factors and clinical outcomes, because of the complex and reciprocal relationships between DM and HCC. To circumvent this weakness related to our study, we conducted the analysis with numerous HCC-related risk factors and demographics on the retrospective review study of the patients with benign liver diseases and HCC. Indeed, the positive correlation risk factors indicated by our analysis, including male gender (OR=5.42, p<0.0001), age (OR=1.056, p<0.0001), HBsAg (OR=14.8, p<0.0001), HCV (OR=6.46, p<0.0001), and cirrhosis (OR=21.8, p<0.0001) (Table 3), were well consistent with the established conclusions [13,25,26,30,31]. This validated the accuracy of our analysis with our retrospective hospital-based cases in predicting the relationship between DM and HCC.

A recent study showed that, the prevalence of diabetes in China is approximately 9% for age 40-50, and 14% for age 50-60. These percentages were calculated based on both diagnosed diabetes cases and previously unreported cases identified by oral glucose tolerance test (GTT) [32]. Indeed, based on this report, the diabetes prevalence percentages of the diagnosed cases and unreported cases were 4.1% and 6.5%, respectively, among men, and 3.5% and 5.2%, respectively, among women. More recently, another study reported that the diabetes prevalence in China was 3.6% in men and 3.4% in women among the diagnosed cases, and 8.5% in men and 7.7% in women among the previously unreported cases [33]. Because our study was a hospital-based retrospective study, the prevalence of diabetes was calculated based on the diagnosed cases. Among 716 patients who were diagnosed with benign liver diseases, the diabetes percentage was 4.2% (Table 1). In comparison, among 852 patients who were diagnosed with HCC, the percentage of diabetes was 10.6%. In our study, the prevalence of diagnosed diabetes was slightly higher than that previously reported [32,33]. The higher diabetes prevalence in our study may be due to the fact that all the participants had either benign or malignant liver diseases, which is consistent with our conclusion that diabetes is positively correlated with HCC.

The findings of the current study indicate that DM patients in the HBV endemic area, such as China, are vulnerable to HCC development. Development of specific guidelines for the prevention and treatment of HCC needs to consider these findings. Periodical screening HCC in aged male patients with DM or DM patients infected with HBV should be warranted. Such guidelines should outline appropriate and safe treatment to prevent HCC in these patients, with the ultimate goal of preventing progressive liver disease and HCC development. 

## Supporting Information

File S1 **Mantel-Haenszel statistical analysis of the effect of the strata, “Younger” and “Older” age groups.** Tables A, B and C show the odds ratios of entire samples, “Younger” group, and “Older” group, respectively. Both estimates (“Younger” and “Older”) of the odds ratios are lower than the odds ratio based on the entire samples.
(DOCX)
