# Supplementary material for: Diabetes Mellitus Is Associated with Hepatocellular Carcinoma: A Retrospective Case-Control Study in Hepatitis Endemic Area
Source: PLoS One. 2013 Dec 26;8(12):e84776. doi: 10.1371/journal.pone.0084776 (PMC3873428; doi:10.1371/journal.pone.0084776)
Supplement: File S1 — Mantel-Haenszel statistical analysis of the effect of the strata, “Younger” and “Older” age groups. Tables A, B and C show the odds ratios of entire samples, “Younger” group, and “Older” group, respectively. Both estimates (“Younger” and “Older”) of the odds ratios are lower than the odds ratio based on the entire samples. (DOCX) [file pone.0084776.s001.docx]

**File S1**

File S1 legend: Mantel-Haenszel statistical analysis of the effect of the strata, “Younger” and “Older” age groups. Tables A, B and C show the odds ratios of entire samples, “Younger” group, and “Older” group, respectively. Both estimates (“Younger” and “Older”) of the odds ratios are lower than the odds ratio based on the entire samples.

**Table A 2×2 table of Diabetes and HCC for entire participants**

|  |  | HCC | |
| --- | --- | --- | --- |
|  |  | no | yes |
| Diabetes | no | 686 | 762 |
|  | yes | 30 | 90 |

Raw Odds Ratio=(686×90)/(30×762)=2.7

**Table B 2×2 table of Diabetes and HCC for “Younger” group**

|  |  | HCC | |
| --- | --- | --- | --- |
|  |  | no | yes |
| Diabetes | no | 354 | 201 |
|  | yes | 5 | 5 |

Odds Ratio=(354×5)/(5×201)=1.8

Age: Control 38.5 ± 8.6

HCC 41.7 ± 6.7

**Table C 2×2 table of Diabetes and HCC for “Older” group**

|  |  | HCC | |
| --- | --- | --- | --- |
|  |  | no | yes |
| Diabetes | no | 332 | 561 |
|  | yes | 25 | 85 |

Odds Ratio=(332×85)/(561×25)=2.0

Age: Control 59.4 ± 7.2

HCC 62.5 ± 8.2
